# *catena*-Poly[[[aqua­bis­(1*H*-imidazole-κ*N* ^3^)copper(II)]-μ-furan-2,5-di­car­boxylato-κ^2^ *O* ^2^:*O* ^5^] trihydrate]

**DOI:** 10.1107/S1600536812016856

**Published:** 2012-04-21

**Authors:** Ya-Feng Li, Yue Xu, Xiao-Lin Qin, Wen-Yuan Gao, Yue Gao

**Affiliations:** a School of Chemical Engineering, Changchun University of Technology, Changchun 130012, People’s Republic of China

## Abstract

In the title cooridnation polymer, {[Cu(C_6_H_2_O_5_)(C_3_H_4_N_2_)_2_(H_2_O)]·3H_2_O}_*n*_, an infinite chain is formed along [001] by linking of the Cu(C_3_N_2_H_4_)_2_(H_2_O) entities with two bridging monodentate carboxyl­ate groups of two different furan-2,5-dicarboxyl­ate dianions. The geometry of the Cu^2+^ ion is a square-based pyramid with the water atom in the apical position and the ligand O and N atoms in a *trans* orientation. The dihedral angle between the imidazole planes is 83.96 (14)°. O_w_–H⋯O and N_i_–H⋯O (w = water and i = imidazole) hydrogen bonds help to establish the packing.

## Related literature

For related structures and background to coordination polymers and their potential uses, see: Li *et al.* (2012*a* ▶,*b* ▶).
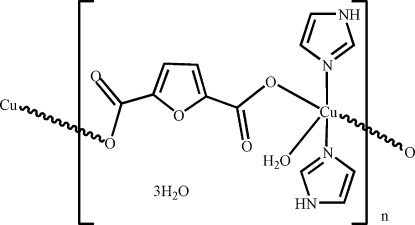


## Experimental

### 

#### Crystal data

[Cu(C_6_H_2_O_5_)(C_3_H_4_N_2_)_2_(H_2_O)]·3H_2_O
*M* *_r_* = 425.85
Monoclinic, 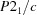

*a* = 7.5725 (15) Å
*b* = 13.339 (3) Å
*c* = 18.881 (5) Å
β = 113.42 (3)°
*V* = 1750.0 (7) Å^3^
*Z* = 4
Mo *K*α radiation
μ = 1.30 mm^−1^
*T* = 293 K
0.59 × 0.38 × 0.26 mm

#### Data collection

Rigaku R-AXIS RAPID diffractometer
Absorption correction: multi-scan (*ABSCOR*; Higashi, 1995 ▶) *T*_min_ = 0.514, *T*_max_ = 0.728
16495 measured reflections
3977 independent reflections
3204 reflections with *I* > 2σ(*I*)
*R*_int_ = 0.059

#### Refinement

*R*[*F* ^2^ > 2σ(*F* ^2^)] = 0.033
*wR*(*F* ^2^) = 0.084
*S* = 1.03
3977 reflections
259 parameters
13 restraints
H atoms treated by a mixture of independent and constrained refinement
Δρ_max_ = 0.57 e Å^−3^
Δρ_min_ = −0.58 e Å^−3^

### 

Data collection: *PROCESS-AUTO* (Rigaku, 1998 ▶); cell refinement: *PROCESS-AUTO*; data reduction: *CrystalStructure* (Rigaku/MSC, 2002 ▶); program(s) used to solve structure: *SHELXS97* (Sheldrick, 2008 ▶); program(s) used to refine structure: *SHELXL97* (Sheldrick, 2008 ▶); molecular graphics: *DIAMOND* (Brandenburg, 2000 ▶); software used to prepare material for publication: *SHELXL97*.

## Supplementary Material

Crystal structure: contains datablock(s) I, global. DOI: 10.1107/S1600536812016856/hb6727sup1.cif

Structure factors: contains datablock(s) I. DOI: 10.1107/S1600536812016856/hb6727Isup2.hkl

Additional supplementary materials:  crystallographic information; 3D view; checkCIF report

## Figures and Tables

**Table 1:** Selected bond lengths (Å)

Cu1—N3	1.9638 (18)
Cu1—N1	1.9826 (17)
Cu1—O4^i^	1.9844 (14)
Cu1—O1	1.9858 (15)
Cu1—O1*W*	2.2797 (17)

Symmetry code: (i) 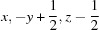.

**Table 2:** Hydrogen-bond geometry (Å, °)

*D*—H⋯*A*	*D*—H	H⋯*A*	*D*⋯*A*	*D*—H⋯*A*
N2—H2*C*⋯O4^ii^	0.86	2.10	2.953 (2)	169
N4—H4*C*⋯O3*W*^iii^	0.86	1.95	2.756 (3)	156
O1*W*—H1*A*⋯O4*W*^iv^	0.87 (2)	2.00 (2)	2.852 (3)	168 (2)
O1*W*—H1*B*⋯O2*W*^v^	0.86 (2)	2.02 (2)	2.861 (3)	169 (3)
O2*W*—H2*A*⋯O5^vi^	0.82 (2)	1.95 (2)	2.756 (2)	166 (3)
O2*W*—H2*B*⋯O2^vii^	0.85 (2)	1.89 (2)	2.739 (2)	172 (2)
O3*W*—H3*A*⋯O1	0.84 (2)	2.22 (2)	3.013 (3)	157 (3)
O3*W*—H3*B*⋯O2*W*	0.81 (2)	1.93 (2)	2.705 (3)	161 (3)
O4*W*—H4*A*⋯O3*W*	0.88 (2)	2.23 (3)	2.878 (4)	130 (3)
O4*W*—H4*B*⋯O2^vii^	0.90 (2)	2.16 (2)	3.052 (3)	171 (3)

Symmetry codes: (ii) 
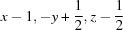
; (iii) 
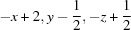
; (iv) 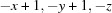; (v) 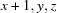; (vi) 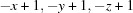; (vii) 
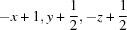
.

**Fig. 1.**
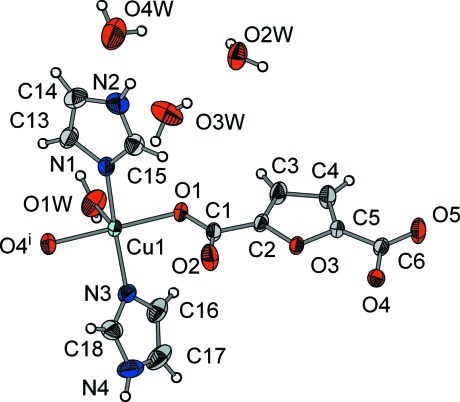
The unit cell of (I), showing displacement ellipsoids at the 50% probability level. [Symmetry codes: (i) 1) x, 0.5 - y, -0.5 + z.]

**Fig. 2.**
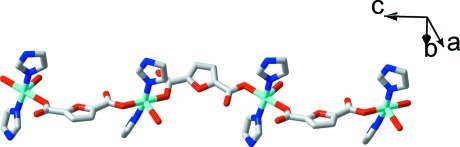
The stick plot of (I), displaying the infinite chain along [001] direction formed by linking the Cu with two monodentate carboxyls of two different furan-2,5-dicarboxylate.

**Fig. 3.**
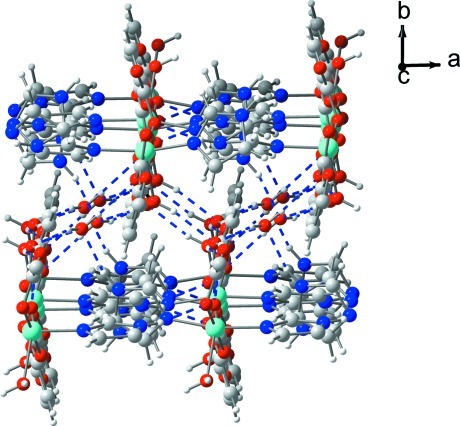
The ball-stick packing diagram of (I). Owater–H···O and Nimidazole–H···O H-bonding interactions together link the adjcent chains to supermolecular net.

## supplementary crystallographic information

### Comment

Recently, we utilized furan-2,5-dicarboxyl acid as the ligand to construct coordination polymers (Li, *et al.*, 2012a,b). As an extension of this work, a new chainlike compound, [Cu(C_3_N_2_H_4_)_2_(H_2_O)(C_6_H_2_O_5_)]^.^3H_2_O (I), is now described.

The asymmetric unit of (I) is consisted of one Cu(II) cation, one furan-2,5-dicarboxylate anion, two imidazole molecules and four water molecules – one coordinated water molecule and three crystallizated water molecules (Fig.1). Cu cation is coordinated by two carboxylate O atoms (d_Cu1–O_ of 1.985 (8) Å and 1.986 (8) Å) from different furan-2,5-dicarboxylate, two *trans*-arranged imidazoles (d_Cu1–N_ of 1.964 (4) Å and 1.984 (4) Å) and one coordinated water molecule (d_Cu1–O1W_ = 2.280 (5) Å) which locates at the axial position, exhibiting distorted pyramid. Two monodentate coordinated carboxyls of furan-2,5-dicarboxylate involve in the formation of infinite chain. The furan-2,5-dicarboxylate shows bridging µ^1^,µ^1^ coordinated mode.

Cu cations are linked by two monodentate carboxylate of different furan-2,5-dicarboxylate to give rise to an infinite chain (Fig.2). O_water_–H···O and N_imidazole_–H···O H-bonding interactions together link the adjcent chains to supermolecular net. (Fig.3).

### Experimental

In a typically synthesized route of (I), furan-2,5-dicarboxyl acid (0.0156 g, 0.10 mmol), Cu(NO_3_)_2_^.^2.5H_2_O (0.0233 g, 0.10 mmol), and C_3_N_2_H_4_ (0.020, 0.30 mmol) and NaOH (0.004, 0.10 mmol) were dissolved in water (5 ml, 278 mmol) under stirring. The mixture with molar ratio of 1 (furan-2,5-dicarboxyl acid): 1 (Cu(NO_3_)_2_^.^2.5H_2_O): 3 (C_3_N_2_H_4_): 1 NaOH: 2780 H_2_O was layed under room temperature for 2 days. The blue block product was collected as a single phase.

### Refinement

Water H atoms were located in a difference Fourier map and refined with O—H = 0.81–0.90 Å and *U*_iso_(H) = 1.2Ueq(O). The carbon H-atoms and nitrogen H-atoms were placed in calculated positions (C—H = 0.93 Å and N—H = 0.86 Å) and were included in the refinement in the riding-model approximation, with *U*_iso_(H) = 1.2Ueq(C).

### Crystal data

**Table:**

[Cu(C_6_H_2_O_5_)(C_3_H_4_N_2_)_2_(H_2_O)]·3H_2_O	*F*(000) = 876
*M**_r_* = 425.85	*D*_x_ = 1.616 Mg m^−^^3^
Monoclinic, *P*2_1_/*c*	Mo *K*α radiation, λ = 0.71073 Å
Hall symbol: -P 2ybc	Cell parameters from 2000 reflections
*a* = 7.5725 (15) Å	θ = 3.1–27.5°
*b* = 13.339 (3) Å	µ = 1.30 mm^−^^1^
*c* = 18.881 (5) Å	*T* = 293 K
β = 113.42 (3)°	Block, blue
*V* = 1750.0 (7) Å^3^	0.59 × 0.38 × 0.26 mm
*Z* = 4	

### Data collection

**Table:**

Rigaku R-AXIS RAPID diffractometer	3977 independent reflections
Radiation source: fine-focus sealed tube	3204 reflections with *I* > 2σ(*I*)
Graphite monochromator	*R*_int_ = 0.059
Detector resolution: 10.00 pixels mm^-1^	θ_max_ = 27.5°, θ_min_ = 3.1°
ω scans	*h* = −9→9
Absorption correction: multi-scan (*ABSCOR*; Higashi, 1995)	*k* = −17→17
*T*_min_ = 0.514, *T*_max_ = 0.728	*l* = −24→24
16495 measured reflections	

### Refinement

**Table:**

Refinement on *F*^2^	Primary atom site location: structure-invariant direct methods
Least-squares matrix: full	Secondary atom site location: difference Fourier map
*R*[*F*^2^ > 2σ(*F*^2^)] = 0.033	Hydrogen site location: inferred from neighbouring sites
*wR*(*F*^2^) = 0.084	H atoms treated by a mixture of independent and constrained refinement
*S* = 1.03	*w* = 1/[σ^2^(*F*_o_^2^) + (0.0436*P*)^2^] where *P* = (*F*_o_^2^ + 2*F*_c_^2^)/3
3977 reflections	(Δ/σ)_max_ < 0.001
259 parameters	Δρ_max_ = 0.57 e Å^−^^3^
13 restraints	Δρ_min_ = −0.58 e Å^−^^3^

### Special details

**Table:**

Geometry. All e.s.d.'s (except the e.s.d. in the dihedral angle between two l.s. planes) are estimated using the full covariance matrix. The cell e.s.d.'s are taken into account individually in the estimation of e.s.d.'s in distances, angles and torsion angles; correlations between e.s.d.'s in cell parameters are only used when they are defined by crystal symmetry. An approximate (isotropic) treatment of cell e.s.d.'s is used for estimating e.s.d.'s involving l.s. planes.
Refinement. Refinement of *F*^2^ against ALL reflections. The weighted *R*-factor *wR* and goodness of fit *S* are based on *F*^2^, conventional *R*-factors *R* are based on *F*, with *F* set to zero for negative *F*^2^. The threshold expression of *F*^2^ > σ(*F*^2^) is used only for calculating *R*-factors(gt) *etc*. and is not relevant to the choice of reflections for refinement. *R*-factors based on *F*^2^ are statistically about twice as large as those based on *F*, and *R*- factors based on ALL data will be even larger.

### Fractional atomic coordinates and isotropic or equivalent isotropic displacement parameters (Å^2^)

**Table:**

	*x*	*y*	*z*	*U*_iso_*/*U*_eq_	
Cu1	0.80735 (3)	0.279641 (17)	0.187220 (13)	0.02352 (9)	
O1	0.7606 (2)	0.35512 (11)	0.26878 (8)	0.0341 (3)	
O2	0.8142 (3)	0.22327 (10)	0.34881 (10)	0.0417 (4)	
O3	0.81345 (18)	0.34055 (9)	0.46325 (7)	0.0244 (3)	
O4	0.85663 (19)	0.29295 (10)	0.60515 (8)	0.0293 (3)	
O5	0.7608 (3)	0.44502 (11)	0.62582 (9)	0.0440 (4)	
N1	0.5261 (2)	0.27626 (12)	0.12482 (10)	0.0289 (4)	
N2	0.2161 (3)	0.26486 (15)	0.08964 (13)	0.0416 (5)	
H2C	0.1064	0.2561	0.0923	0.050*	
N3	1.0749 (2)	0.25501 (13)	0.25855 (10)	0.0304 (4)	
N4	1.3374 (3)	0.17360 (18)	0.32566 (13)	0.0510 (6)	
H4C	1.4207	0.1260	0.3413	0.061*	
C1	0.7868 (3)	0.31396 (15)	0.33307 (12)	0.0282 (4)	
C2	0.7783 (3)	0.38283 (14)	0.39338 (11)	0.0246 (4)	
C3	0.7261 (3)	0.47994 (15)	0.39320 (13)	0.0367 (5)	
H3	0.6959	0.5251	0.3525	0.044*	
C4	0.7265 (3)	0.49939 (15)	0.46681 (12)	0.0359 (5)	
H4	0.6956	0.5597	0.4839	0.043*	
C5	0.7806 (3)	0.41319 (14)	0.50774 (11)	0.0248 (4)	
C6	0.8006 (3)	0.38347 (15)	0.58597 (11)	0.0259 (4)	
C13	0.4381 (3)	0.29424 (18)	0.04730 (13)	0.0402 (5)	
H13	0.5004	0.3088	0.0149	0.048*	
C14	0.2447 (3)	0.28732 (19)	0.02537 (16)	0.0479 (6)	
H14	0.1507	0.2963	−0.0241	0.058*	
C15	0.3867 (3)	0.25861 (16)	0.14793 (14)	0.0352 (5)	
H15	0.4053	0.2437	0.1985	0.042*	
C16	1.1938 (3)	0.3177 (2)	0.31533 (13)	0.0415 (5)	
H16	1.1667	0.3834	0.3240	0.050*	
C17	1.3568 (3)	0.2670 (2)	0.35633 (16)	0.0556 (8)	
H17	1.4627	0.2915	0.3979	0.067*	
C18	1.1672 (3)	0.16913 (18)	0.26737 (14)	0.0396 (5)	
H18	1.1190	0.1128	0.2367	0.048*	
O1W	0.8545 (3)	0.42683 (13)	0.13588 (11)	0.0493 (4)	
H1A	0.847 (4)	0.423 (2)	0.0890 (10)	0.059*	
H1B	0.963 (3)	0.454 (2)	0.1640 (13)	0.059*	
O2W	0.1888 (3)	0.54338 (12)	0.22157 (10)	0.0456 (4)	
H2A	0.224 (4)	0.5465 (18)	0.2687 (9)	0.055*	
H2B	0.182 (3)	0.6015 (13)	0.2018 (13)	0.055*	
O3W	0.4606 (3)	0.49932 (15)	0.16719 (15)	0.0676 (6)	
H3A	0.552 (3)	0.458 (2)	0.1840 (19)	0.081*	
H3B	0.387 (4)	0.500 (2)	0.1888 (18)	0.081*	
O4W	0.2270 (4)	0.5961 (2)	0.02425 (14)	0.0892 (8)	
H4A	0.264 (5)	0.5371 (16)	0.046 (2)	0.107*	
H4B	0.202 (5)	0.636 (2)	0.0576 (18)	0.107*	

### Atomic displacement parameters (Å^2^)

**Table:**

	*U*^11^	*U*^22^	*U*^33^	*U*^12^	*U*^13^	*U*^23^
Cu1	0.02336 (13)	0.03122 (14)	0.01630 (13)	0.00077 (9)	0.00822 (9)	−0.00176 (9)
O1	0.0403 (8)	0.0447 (8)	0.0176 (7)	0.0118 (7)	0.0117 (6)	0.0003 (6)
O2	0.0651 (11)	0.0320 (8)	0.0369 (10)	−0.0002 (8)	0.0297 (9)	−0.0051 (6)
O3	0.0307 (7)	0.0262 (6)	0.0169 (7)	0.0018 (6)	0.0101 (6)	−0.0004 (5)
O4	0.0310 (7)	0.0353 (7)	0.0256 (8)	0.0065 (6)	0.0156 (6)	0.0080 (6)
O5	0.0704 (11)	0.0414 (8)	0.0265 (9)	0.0090 (8)	0.0258 (8)	−0.0022 (6)
N1	0.0247 (8)	0.0383 (9)	0.0245 (9)	−0.0025 (7)	0.0107 (7)	−0.0036 (7)
N2	0.0247 (9)	0.0530 (11)	0.0484 (13)	−0.0066 (8)	0.0159 (8)	−0.0058 (9)
N3	0.0260 (8)	0.0385 (9)	0.0251 (9)	−0.0011 (7)	0.0084 (7)	0.0004 (7)
N4	0.0297 (10)	0.0728 (15)	0.0469 (14)	0.0107 (10)	0.0115 (9)	0.0212 (12)
C1	0.0269 (10)	0.0379 (10)	0.0208 (10)	−0.0006 (8)	0.0103 (8)	−0.0050 (8)
C2	0.0269 (9)	0.0307 (9)	0.0162 (9)	−0.0005 (8)	0.0085 (8)	0.0018 (7)
C3	0.0564 (14)	0.0320 (10)	0.0247 (11)	0.0066 (10)	0.0193 (10)	0.0067 (8)
C4	0.0568 (13)	0.0278 (10)	0.0265 (11)	0.0063 (10)	0.0201 (10)	0.0009 (8)
C5	0.0267 (9)	0.0292 (9)	0.0192 (10)	0.0013 (8)	0.0100 (8)	−0.0020 (7)
C6	0.0245 (9)	0.0337 (10)	0.0194 (10)	−0.0016 (8)	0.0087 (8)	−0.0007 (8)
C13	0.0312 (11)	0.0641 (15)	0.0247 (12)	−0.0038 (10)	0.0104 (9)	0.0001 (10)
C14	0.0299 (11)	0.0687 (17)	0.0375 (15)	−0.0005 (11)	0.0051 (10)	−0.0023 (12)
C15	0.0337 (11)	0.0407 (11)	0.0338 (12)	−0.0052 (9)	0.0161 (10)	0.0003 (9)
C16	0.0406 (12)	0.0503 (13)	0.0297 (12)	−0.0143 (10)	0.0100 (10)	−0.0041 (10)
C17	0.0308 (12)	0.097 (2)	0.0308 (14)	−0.0189 (13)	0.0033 (10)	0.0092 (14)
C18	0.0300 (11)	0.0509 (13)	0.0406 (14)	0.0020 (10)	0.0168 (10)	0.0045 (11)
O1W	0.0618 (11)	0.0474 (9)	0.0358 (10)	−0.0173 (9)	0.0164 (9)	0.0027 (8)
O2W	0.0711 (12)	0.0367 (8)	0.0329 (10)	−0.0039 (8)	0.0247 (9)	0.0019 (7)
O3W	0.0549 (12)	0.0521 (11)	0.0975 (19)	0.0156 (10)	0.0322 (12)	−0.0010 (11)
O4W	0.126 (2)	0.0902 (17)	0.0541 (16)	0.0284 (17)	0.0381 (15)	0.0092 (13)

### Geometric parameters (Å, º)

**Table:**

Cu1—N3	1.9638 (18)	C2—C3	1.354 (3)
Cu1—N1	1.9826 (17)	C3—C4	1.413 (3)
Cu1—O4^i^	1.9844 (14)	C3—H3	0.9300
Cu1—O1	1.9858 (15)	C4—C5	1.355 (3)
Cu1—O1W	2.2797 (17)	C4—H4	0.9300
O1—C1	1.274 (2)	C5—C6	1.478 (3)
O2—C1	1.243 (2)	C13—C14	1.357 (3)
O3—C2	1.360 (2)	C13—H13	0.9300
O3—C5	1.368 (2)	C14—H14	0.9300
O4—C6	1.283 (2)	C15—H15	0.9300
O5—C6	1.229 (2)	C16—C17	1.349 (3)
N1—C15	1.314 (3)	C16—H16	0.9300
N1—C13	1.367 (3)	C17—H17	0.9300
N2—C15	1.326 (3)	C18—H18	0.9300
N2—C14	1.349 (3)	O1W—H1A	0.866 (16)
N2—H2C	0.8600	O1W—H1B	0.858 (16)
N3—C18	1.318 (3)	O2W—H2A	0.822 (16)
N3—C16	1.375 (3)	O2W—H2B	0.853 (15)
N4—C18	1.322 (3)	O3W—H3A	0.843 (17)
N4—C17	1.357 (4)	O3W—H3B	0.808 (17)
N4—H4C	0.8600	O4W—H4A	0.884 (18)
C1—C2	1.484 (3)	O4W—H4B	0.900 (18)
			
N3—Cu1—N1	167.24 (7)	C2—C3—C4	106.66 (18)
N3—Cu1—O4^i^	89.48 (7)	C2—C3—H3	126.7
N1—Cu1—O4^i^	90.91 (7)	C4—C3—H3	126.7
N3—Cu1—O1	90.35 (7)	C5—C4—C3	106.69 (18)
N1—Cu1—O1	89.54 (7)	C5—C4—H4	126.7
O4^i^—Cu1—O1	178.68 (6)	C3—C4—H4	126.7
N3—Cu1—O1	90.35 (7)	C4—C5—O3	109.77 (17)
N1—Cu1—O1	89.54 (7)	C4—C5—C6	133.28 (18)
O4^i^—Cu1—O1	178.68 (6)	O3—C5—C6	116.88 (16)
N3—Cu1—O1W	98.18 (7)	O5—C6—O4	126.13 (19)
N1—Cu1—O1W	94.58 (7)	O5—C6—C5	118.68 (18)
O4^i^—Cu1—O1W	88.76 (6)	O4—C6—C5	115.19 (17)
O1—Cu1—O1W	89.96 (7)	C14—C13—N1	108.8 (2)
O1—Cu1—O1W	89.96 (7)	C14—C13—H13	125.6
C1—O1—Cu1	120.82 (13)	N1—C13—H13	125.6
C2—O3—C5	106.77 (14)	N2—C14—C13	106.3 (2)
C6—O4—Cu1^ii^	122.40 (13)	N2—C14—H14	126.9
C15—N1—C13	105.82 (18)	C13—C14—H14	126.9
C15—N1—Cu1	128.44 (16)	N1—C15—N2	111.1 (2)
C13—N1—Cu1	125.73 (15)	N1—C15—H15	124.5
C15—N2—C14	107.98 (19)	N2—C15—H15	124.5
C15—N2—H2C	126.0	C17—C16—N3	108.0 (2)
C14—N2—H2C	126.0	C17—C16—H16	126.0
C18—N3—C16	106.23 (19)	N3—C16—H16	126.0
C18—N3—Cu1	125.66 (15)	C16—C17—N4	107.2 (2)
C16—N3—Cu1	127.62 (16)	C16—C17—H17	126.4
C18—N4—C17	107.5 (2)	N4—C17—H17	126.4
C18—N4—H4C	126.2	N3—C18—N4	111.0 (2)
C17—N4—H4C	126.2	N3—C18—H18	124.5
O2—C1—O1	126.51 (19)	N4—C18—H18	124.5
O2—C1—O1	126.51 (19)	Cu1—O1W—H1A	115.2 (18)
O2—C1—C2	118.23 (18)	Cu1—O1W—H1B	111.8 (19)
O1—C1—C2	115.25 (18)	H1A—O1W—H1B	109 (2)
O1—C1—C2	115.25 (18)	H2A—O2W—H2B	111 (2)
C3—C2—O3	110.09 (17)	H3A—O3W—H3B	117 (3)
C3—C2—C1	133.77 (19)	H4A—O4W—H4B	108 (2)
O3—C2—C1	115.85 (16)		
			
N3—Cu1—O1—O1	0.00 (17)	O2—C1—C2—C3	−169.3 (2)
N1—Cu1—O1—O1	0.00 (17)	O1—C1—C2—C3	9.3 (3)
N3—Cu1—O1—C1	−57.95 (16)	O1—C1—C2—C3	9.3 (3)
N1—Cu1—O1—C1	109.29 (16)	O2—C1—C2—O3	3.8 (3)
O1—Cu1—O1—C1	0 (100)	O1—C1—C2—O3	−177.51 (16)
O1W—Cu1—O1—C1	−156.13 (16)	O1—C1—C2—O3	−177.51 (16)
N3—Cu1—N1—C15	46.9 (4)	O3—C2—C3—C4	−0.7 (3)
O4^i^—Cu1—N1—C15	138.59 (19)	C1—C2—C3—C4	172.8 (2)
O1—Cu1—N1—C15	−42.66 (19)	C2—C3—C4—C5	0.5 (3)
O1—Cu1—N1—C15	−42.66 (19)	C3—C4—C5—O3	−0.2 (2)
O1W—Cu1—N1—C15	−132.59 (19)	C3—C4—C5—C6	−177.0 (2)
N3—Cu1—N1—C13	−134.4 (3)	C2—O3—C5—C4	−0.2 (2)
O4^i^—Cu1—N1—C13	−42.69 (18)	C2—O3—C5—C6	177.16 (16)
O1—Cu1—N1—C13	136.07 (18)	Cu1^ii^—O4—C6—O5	21.2 (3)
O1—Cu1—N1—C13	136.07 (18)	Cu1^ii^—O4—C6—C5	−157.71 (13)
O1W—Cu1—N1—C13	46.14 (19)	C4—C5—C6—O5	0.9 (4)
N1—Cu1—N3—C18	46.9 (4)	O3—C5—C6—O5	−175.63 (17)
O4^i^—Cu1—N3—C18	−44.92 (18)	C4—C5—C6—O4	179.9 (2)
O1—Cu1—N3—C18	136.40 (19)	O3—C5—C6—O4	3.4 (3)
O1—Cu1—N3—C18	136.40 (19)	C15—N1—C13—C14	0.3 (3)
O1W—Cu1—N3—C18	−133.59 (18)	Cu1—N1—C13—C14	−178.68 (16)
N1—Cu1—N3—C16	−124.0 (3)	C15—N2—C14—C13	0.0 (3)
O4^i^—Cu1—N3—C16	144.21 (19)	N1—C13—C14—N2	−0.2 (3)
O1—Cu1—N3—C16	−34.47 (19)	C13—N1—C15—N2	−0.3 (3)
O1—Cu1—N3—C16	−34.47 (19)	Cu1—N1—C15—N2	178.64 (14)
O1W—Cu1—N3—C16	55.5 (2)	C14—N2—C15—N1	0.2 (3)
O1—O1—C1—O2	0.00 (14)	C18—N3—C16—C17	0.7 (3)
Cu1—O1—C1—O2	−11.2 (3)	Cu1—N3—C16—C17	173.02 (16)
Cu1—O1—C1—O1	0 (100)	N3—C16—C17—N4	−0.7 (3)
O1—O1—C1—C2	0.0 (2)	C18—N4—C17—C16	0.4 (3)
Cu1—O1—C1—C2	170.28 (12)	C16—N3—C18—N4	−0.5 (3)
C5—O3—C2—C3	0.5 (2)	Cu1—N3—C18—N4	−172.96 (15)
C5—O3—C2—C1	−174.22 (16)	C17—N4—C18—N3	0.0 (3)

Symmetry codes: (i) *x*, −*y*+1/2, *z*−1/2; (ii) *x*, −*y*+1/2, *z*+1/2.

### Hydrogen-bond geometry (Å, º)

**Table:**

*D*—H···*A*	*D*—H	H···*A*	*D*···*A*	*D*—H···*A*
N2—H2*C*···O4^iii^	0.86	2.10	2.953 (2)	169
N4—H4*C*···O3*W*^iv^	0.86	1.95	2.756 (3)	156
O1*W*—H1*A*···O4*W*^v^	0.87 (2)	2.00 (2)	2.852 (3)	168 (2)
O1*W*—H1*B*···O2*W*^vi^	0.86 (2)	2.02 (2)	2.861 (3)	169 (3)
O2*W*—H2*A*···O5^vii^	0.82 (2)	1.95 (2)	2.756 (2)	166 (3)
O2*W*—H2*B*···O2^viii^	0.85 (2)	1.89 (2)	2.739 (2)	172 (2)
O3*W*—H3*A*···O1	0.84 (2)	2.22 (2)	3.013 (3)	157 (3)
O3*W*—H3*B*···O2*W*	0.81 (2)	1.93 (2)	2.705 (3)	161 (3)
O4*W*—H4*A*···O3*W*	0.88 (2)	2.23 (3)	2.878 (4)	130 (3)
O4*W*—H4*B*···O2^viii^	0.90 (2)	2.16 (2)	3.052 (3)	171 (3)

Symmetry codes: (iii) *x*−1, −*y*+1/2, *z*−1/2; (iv) −*x*+2, *y*−1/2, −*z*+1/2; (v) −*x*+1, −*y*+1, −*z*; (vi) *x*+1, *y*, *z*; (vii) −*x*+1, −*y*+1, −*z*+1; (viii) −*x*+1, *y*+1/2, −*z*+1/2.
